# Deep Investigation of *Arabidopsis thaliana* Junk DNA Reveals a Continuum between Repetitive Elements and Genomic Dark Matter

**DOI:** 10.1371/journal.pone.0094101

**Published:** 2014-04-07

**Authors:** Florian Maumus, Hadi Quesneville

**Affiliations:** UR1164 URGI - Research Unit in Genomics-Info, INRA, Versailles, France; Georgia Institute of Technology, United States of America

## Abstract

Eukaryotic genomes contain highly variable amounts of DNA with no apparent function. This so-called junk DNA is composed of two components: repeated and repeat-derived sequences (together referred to as the repeatome), and non-annotated sequences also known as genomic dark matter. Because of their high duplication rates as compared to other genomic features, transposable elements are predominant contributors to the repeatome and the products of their decay is thought to be a major source of genomic dark matter. Determining the origin and composition of junk DNA is thus important to help understanding genome evolution as well as host biology. In this study, we have used a combination of tools enabling to show that the repeatome from the small and reducing *A. thaliana* genome is significantly larger than previously thought. Furthermore, we present the concepts and results from a series of innovative approaches suggesting that a significant amount of the *A. thaliana* dark matter is of repetitive origin. As a tentative standard for the community, we propose a deep compendium annotation of the *A. thaliana* repeatome that may help addressing farther genome evolution as well as transcriptional and epigenetic regulation in this model plant.

## Introduction

The repeatome can be defined as the complement of repeated sequences in a genome. The eukaryotic repeatomes typically comprise variable amounts of multiple components including transposable elements (TEs) and endogenous viruses, simple sequence repeats, segmental duplications, ribosomal DNA and other ribozymes, multi-copy gene families, retropseudogenes, as well as highly conserved and repeated protein domains. The repeatome is paramount in the “C-value paradox” [1] which states that genome size does not correlate with apparent organismal complexity and suggests that, besides functional sequences including host genes and regulatory sequences, eukaryotic genomes comprise a highly variable amount of DNA with no evident function that is predominant in haploid genome size variation. As a function in host biology remains elusive (or putative for most), this superfluous or unnecessary DNA was called “junk” DNA [2]. Junk DNA can be split into two major components: repeated sequences [3], and genomic dark matter (as defined by the not annotated sequences). TEs comprise a wide variety of mobile genetic entities that are able to move from one place to another in a host genome. Because of their relative high duplication rate as compared to other genomic components, TEs are typically predominant contributors to eukaryotic repeatomes [4]. TEs illustrate the junk DNA concept as they can accumulate in host genomes with no evident beneficial function in host biology; and in this aspect can be viewed as selfish genomic parasites [5] [6]. In addition, their taxonomic distributions suggest that most major types of TEs were present in the last eukaryotic common ancestor. This indicates that TEs have been jumping around eukaryotic host genes for over a billion years, thereby suggesting that a significant fraction of the genomic dark matter could derive from the gigantic amounts of TE genetic material that have been sculpting genomes over evolutionary times [7].

Besides enlightening the history of genome evolution, the characterization of junk DNA is of primary importance for understanding host biology and species evolution. Indeed, while one could accept that a synthetic eukaryotic genome would conceptually not require the incorporation of junk DNA in order to be functional, a colossal amount of evidence suggests that TEs are supreme players in the evolution and adaptation of natural species. After decades of research in a variety of species, TEs collectively appear to play roles in countless layers of host biology and evolution including in epigenetic regulation, the elaboration of transcriptional networks, genome rearrangements, chromosome sculpting (including centromere organization), gene duplication, exon shuffling, and so on [8] [9] [10] [11].

Altogether, while there is a fundamental interest in deciphering the nature and origins of junk DNA, the thorough detection of repeated and repeat-derived sequences represents species-specific methodological challenges. While TEs have been extensively characterized manually in some model species, the important size and number of sequenced eukaryotic genomes has driven the development of *de novo* and automated computational solutions. There are three main approaches for the computational identification of repeated sequences *i.e*. k-mer-based, similarity-based, or structure-based, which respectively rely on the search for: frequent words of size k (k-mers), high scoring pairs (HSPs) by all-by-all genome comparison, and structural features that are characteristic of TEs [12] [13]. Several widely distributed programs such as RepeatScout [14], RepeatModeler [15] and REPET [16] build workflows using these strategies for the construction of consensus sequences that aim at representing as much as possible the repeatome diversity. However, the inherent differences between *de novo* repeat finding programs supposes that they are empirically complementary [12] [13] to an extent that likely correlates with repeatome complexity. Nevertheless, the majority of the eukaryotic repeatomes are resolved using one or another program and the potential benefit of their combination remains to be assessed. Furthermore, while conservative approaches are virtuous for the establishment of standards, some more exploratory strategies may yield unanticipated results that shed light on the nature and history of junk DNA. For instance, using an innovative approach [17], de Koning et al. have recently proposed that at least two-thirds of the human genome is made of repeated and repeat-derived sequences [18], thereby beating back the contribution of the dark matter complement. Beyond reinforcing the idea that repeatome and dark matter to a large extent represent the extremes of a continuum, this study also illustrates the need for exploratory routes towards improved characterization of junk complements.

As experimental model we chose the model plant *Arabidopsis thaliana* (accession Col-0) [19] because of its small (119 Mb) genome size and its long history of manual curation providing a high quality genomic sequence and annotation. In addition, we have recently proposed that most of the *A. thaliana* repeatome likely derives from ancestral bursts that occurred 10–20 mya (Maumus and Quesneville, unpublished data), suggesting that some of the dark matter in the *A. thaliana* genome is repeat-derived but remains beyond detection using conservative approaches. In this study, we have assessed the complementarity of several *de novo* repeat-finding programs and found that their combination increases genome coverage by at least 20% as compared to annotations using a single program. Furthermore, we performed several independent computational experiments that enable to annotate substantial amounts of *A. thaliana* dark matter and suggest that the repeatome contributes about one third of the genome.

## Results

### Different programs are complementary for repeatome detection

**Figure 1:**
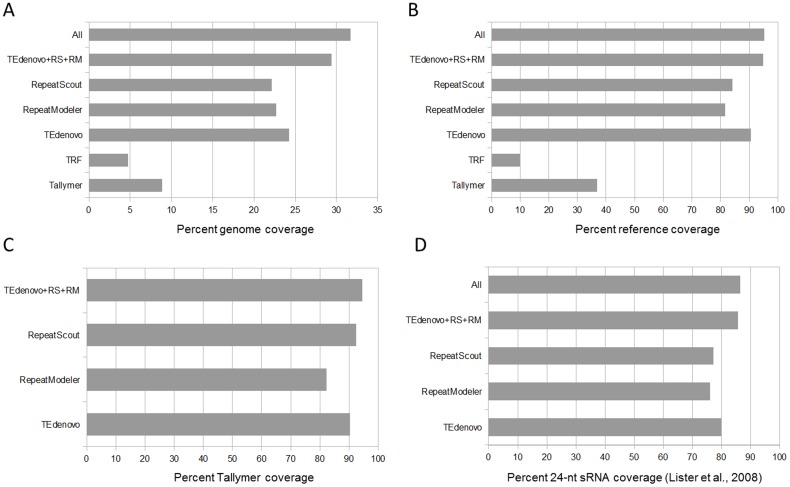
Benefits of the combined approach. The coverage of the genome (A), reference set (B), Tallymer set (C), and 24-nt sRNA map (D) by annotation sets from different programs, the non-redundant combination of annotations from all tools (All), and the non-redundant combination of annotations from TEdenovo, RepeatScout, and RepeatModeler (TEdenovo + RS + RM).

We constructed libraries of consensus sequences by employing the programs called RepeatScout [14], RepeatModeler [15] (hereafter referred to as RS and RM, respectively), and TEdenovo from the REPET package [16] on the *A. thaliana* Col-0 genome. Each library was used separately as input for genome annotation using the TEannot pipeline which is also included in the REPET package (see Methods). In addition, we used the Tallymer and Tandem Repeat Finder (TRF) programs in order to identify more specifically the sets of short perfect repeats and tandem (simple) repeats, respectively. Interestingly, while compared to other tools the genome annotation using the consensus sequences from TEdenovo yields the highest genome coverage (Figure 1A), the combination of all the annotation sets covers ∼32% of the *A. thaliana* genome, i.e. a significant increase as compared to the well accepted approx. 23% repeat content in *A. thaliana* [16]. More specifically, while TEdenovo alone enables the most extensive (∼90%) coverage of the reference annotations (see Methods), the combination of all tools attains 95% coverage. When not taking into account the contribution of the annotations from TRF and Tallymer, the coverage of the reference annotations holds at 95% with 29.4% (35 Mb) genome coverage (Figure 1B). In contrast when comparing the ability of RS, RM, and TEdenovo to detect the annotations generated by Tallymer, we found that RS performed best, showing that consensus sequences generated by the latter program are better representatives of the perfect repeats detected by Tallymer (Figure 1C). Nevertheless, the combination of the RS, RM, and TEdenovo annotations covers over 94% of the Tallymer annotations, further reinforcing the idea that the combination of these tools enables to detect a wider diversity of the *A. thaliana* repeatome. As another type of indicator of sensitivity we used the mapping coordinates of a set of 24-nucleotide (nt) small RNA (sRNA) [20]. Indeed, in plants, 24-nt sRNAs are known to map almost exclusively to repeated sequences as they play an important role in epigenetic silencing [21] [22] [23]. Their mapping positions on the genome can therefore serve as natural repeat-associated cartography. This set of 24-nt sRNA covers 9.7 Mb of the *A. thaliana* genome, 5% of this amount mapping to host protein-coding sequences (CDS, data not shown). While we found that TEdenovo performs best as lone program by covering 80% of the 24-nt sRNA loci, the combination of RS, RM, and TEdenovo enables to cover 86% of the sRNA map (Figure 1D) which is a substantial enhancement as compared to the 73% coverage with the reference annotations.

**Figure 2:**
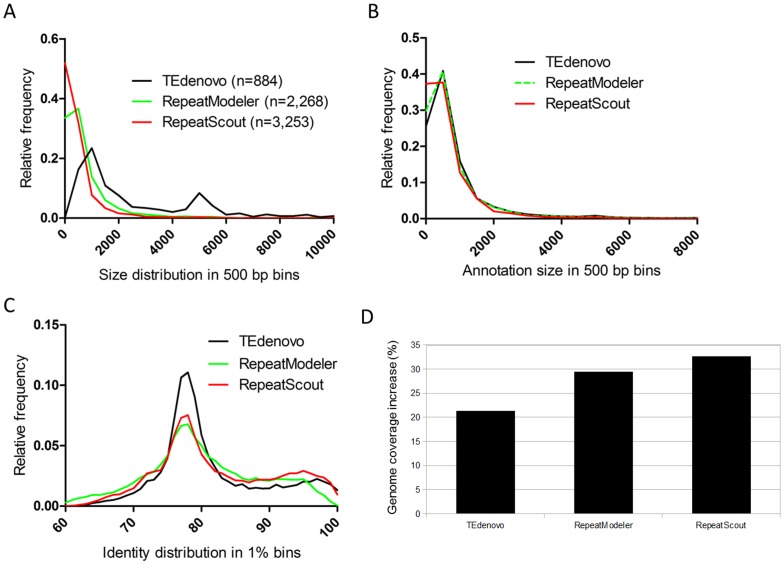
Different repeat detection programs are complementary. (A) Distribution in 500 bp bins of the size of the consensus sequences obtained using different programs. (B) Distribution in 500 bp bins of the size of the annotations obtained using consensus sequences from different programs. (C) Distribution in 1% bins of the identity values between genomic copies and consensus sequences from different programs. (D) Percent increase of genome coverage with the combined TEdenovo, RepeatScout, and RepeatModeler annotations as compared to annotations from each program separately.

When comparing the length of the consensus sequences from the libraries generated by the different repeat-detection programs, we found that the TEdenovo library is enriched in long sequences as compared to those obtained with RS and RM (Figure 2A). In corollary, the annotations obtained using the TEdenovo library are enriched in long copies as compared to those obtained using the RS and RM libraries (Figure 2B, Table 1;). We next measured the identity between each copy and its cognate consensus sequence as a proxy for the measurement of evolutionary distances. The distributions obtained with input libraries from the different programs present overall similar profiles (Figure 2C). Such consistency is likely to testify the same evolutionary history of the repeatome and confirms the presence of an important pool of old repeats in the *A. thaliana* genome (Maumus and Quesneville, unpublished data). In more details though, the RS annotations are relatively enriched in highly similar matches (over 90% identity) especially as compared to the RM annotations, while the TEdenovo ones are relatively enriched in values around 75–80%. Finally, at the chromosome level, the densities of the RS, RM, and TEdenovo annotations seem largely similar, while local variations are also apparent (Figure S1).

**Table 1:** Overall statistics of match sizes using consensus sequences from different programs for genome annotation.

	TEdenovo	RepeatModeler	RepeatScout
25% Percentile	231	187	181
Median	464	327	347
75% Percentile	878	649	707

Overall, while our results suggest the higher quality and sensitivity of the annotations obtained when using the consensus sequences built by TEdenovo, they also highlight the complementarity of the libraries generated by each program. Remarkably, combining the non-redundant annotations from the three programs yields at least 21% increase of genome coverage compared to each program alone (Figure 2D). Hence, while largely overlapping, each of these programs appears to be capable to fetch some specific fractions of the *A. thaliana* repeatome.

### Iterative approach

**Figure 3:**
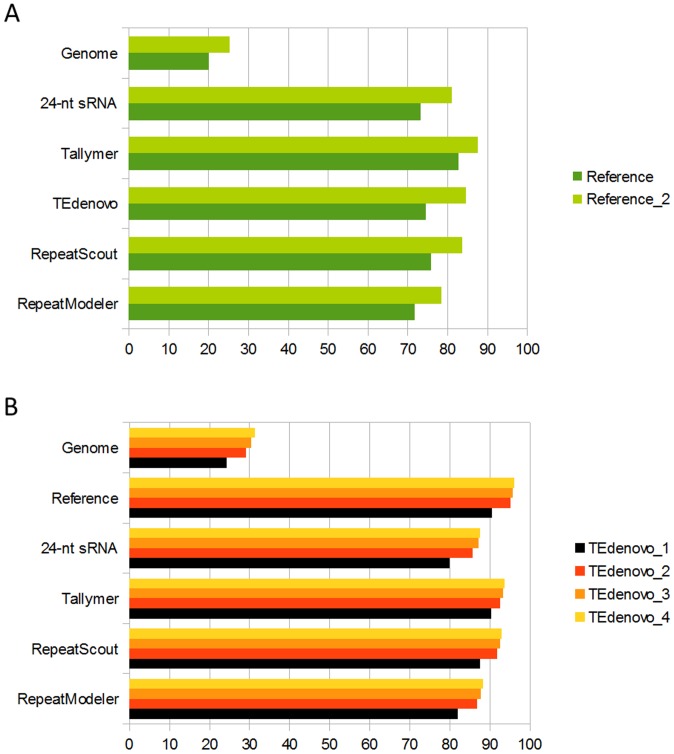
Reiterations beat dark matter back. Coverage of genome and different indicators of sensitivity by annotations from the reiterative approach using the reference sequences (A) and the consensus sequences from TEdenovo (B) as initial library.

Above, we have used consensus sequences generated using conservative parameters in order to detect genomic copies. In principle, the genomic copies that have been identified enclose a greater diversity of the *A. thaliana* repeatome as compared to consensus sequences. We thus reasoned that using genomic copies as probes to search for homolog sequences may enable to detect additional related sequences. The small *A. thaliana* genome enables to perform such an experiment within reasonable computational time. As a benchmark, we have first extracted all the genomic sequences that correspond to the reference annotation set, *i.e*. the copies of previously known repeats. After removing the sequences shorter than 200 bp as empirically less significant, this library was used for genome annotation. As a proof of concept, the annotation set obtained (referred to as Reference_2) covers 30 Mb of the genome as compared to 23.8 Mb with the reference set. This effect is accompanied by significantly increased coverage of a wide set of indicators that serve to represent repeatome complexity including the 24-nt sRNA map introduced previously and the repeat annotations generated above using conservative approaches (Figure 3A).

**Figure 4:**
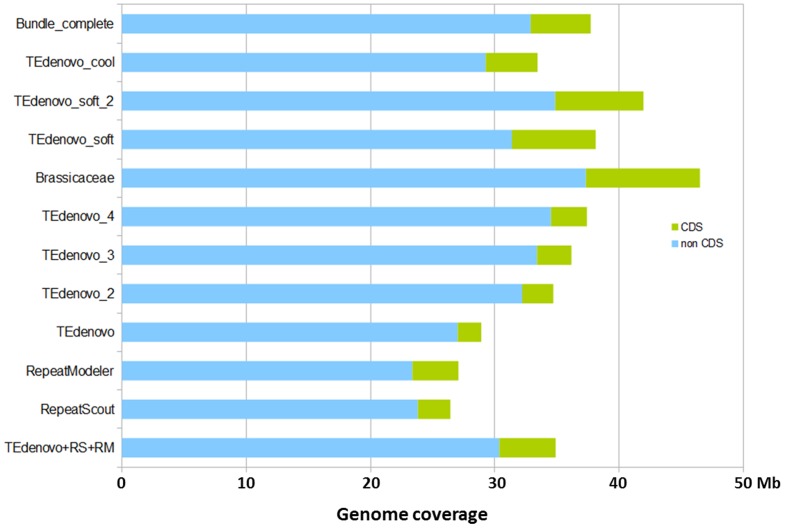
Coverage of the *A. thaliana* genome by the different annotations presented in this work discriminating CDS versus non-CDS contributions. “TEdenovo + RS + RM“ refers to the non-redundant combination of annotations from TEdenovo, RepeatScout, and RepeatModeler.

Having measured the strong potential of such iterative approach for detecting additional repeated or repeat-derived sequences, we next applied it to the TEdenovo annotations since they encompass a greater diversity of the *A. thaliana* repeatome as compared to the reference annotations. This second iteration (hereafter referred to as TEdenovo_2) detected a substantial amount of new annotations as compared to the first one (TEdenovo_1) with a 20% (5.8 Mb) increase of genome coverage (Figure 3B). Qualitatively, over 90% of the supplementary annotations as compared to TEdenovo_1 represent segments of 50 bp or longer (data not shown). In terms of sensitivity, we found that TEdenovo_2 covers 95% of the reference annotation as compared to 90% with TEdenovo_1 (Figure 3B). In addition, TEdenovo_2 covers a significantly greater proportion of the annotations obtained above with other programs thus showing that the reiteration with TEdenovo copies enables to partially palliate the specificities of other programs. Furthermore we found that, while TEdenovo_1 covers 80% of the 24-nt sRNA map, the coverage increases to 86% with TEdenovo_2 (Figure 3B). Altogether our indicators suggest that the reiterative approach enables to substantially increase the sensitivity of repeat detection. Implicitly, the TEdenovo_2 space covers 100% of the TEdenovo_1 annotations (data not shown). When assessing the scattering of the TEdenovo_2 annotations, we found that 81.6% of the copies overlap (by at least 1 bp) with annotations from TEdenovo_1, RM, or RS. These TEdenovo_2 copies collectively contribute 94.9% of the TEdenovo_2 coverage, meaning that the vast majority of the annotations found with TEdenovo_2 are located in regions that have already been defined above as “repeat-positive” (data not shown). Moreover, CDS contribute only 9.9% of the additional annotations which is a rate similar to the ones obtained with conservative approaches (∼10%, see Methods and Figure 4), thus suggesting that the majority of the novel TEdenovo_2 annotations cannot be explained by an increased propensity to target host genes.

We next decided to further address the nature of the supplementary copies that have been found with TEdenovo_2 as compared to TEdenovo_1. We reasoned that assessing directly their aptitude to detect the repeats identified above would be a valuable source of information. In this scope, we have extracted the genomic positions of the new copies by computing the exact differences between TEdenovo_2 and TEdenovo_1 (hereafter referred to as delta_2vs1). After filtering the short annotations (<200 bp), the corresponding sequences were used as input library for genome annotation. Of high significance, we found that this library enables to mask as much as 23 Mb (19.5%) of the genome with a good capacity to detect the repeats that were detected above with conservative approaches. For example, the delta_2vs1 annotations cover 66% and 59% of the reference and TEdenovo annotations, respectively, as well as 56% of the 24-nt sRNA map (data not shown). These results suggest that the supplementary annotations from TEdenovo_2 as compared to TEdenovo_1 are highly representative of the *A. thaliana* repeatome. Furthermore, we found that CDS contribute as little as 5.8% of the delta_2vs1 annotations, thus confirming that the new annotations found by TEdenovo_2 are not to be attributed to a bias towards detecting host genes.

Comforted by these aptitudes, we performed two additional TEdenovo iterations (TEdenovo_3 and TEdenovo_4) by resuming from TEdenovo_2. Remarkably, while we observed a relatively limited increase in genome coverage after these new rounds, we also detect a stepwise increase of all the values of our indicators of sensitivity (Figure 3B). After the fourth round, the coverage of TEdenovo_4 annotations over the reference, RS, RM, and Tallymer annotations reach 96.1, 92.9, 93.6, and 88.8%, respectively. In parallel, the coverage of the 24-nt sRNA map was enhanced to 88% as compared to 80% with TEdenovo_1. Finally, while between TEdenovo_1 and TEdenovo_4 the genome coverage shifts from 28.9 Mb to 37.4 Mb, the CDS coverage rises from 1.9 to 2.9 Mb. Correlatively, CDS contribute 7.7% of the TEdenovo_4 annotations as compared to 6.6% with TEdenovo_1 (Figure 4), thus showing a rather steady host gene contribution over the successive rounds.

**Figure 5:**
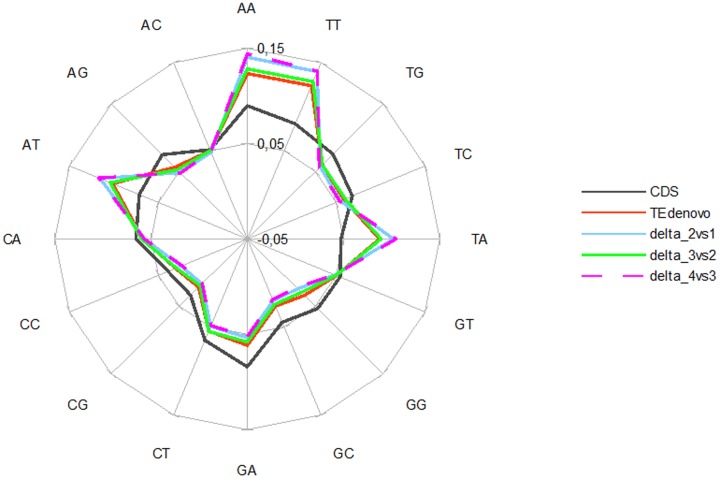
Nature of the annotations from reiteration. Dinucleotide frequencies in the sets of CDS, and TEdenovo annotations, as well as in the annotations detected specifically by the reiterative approach as compared to the previous round (*e.g* delta_2vs1 comprises the difference between TEdnovo_2 and TEdenovo_1).

We further investigated the essence of the supplementary annotations obtained using this reiterative approach. The relative abundance of dinucleotides constitutes a signature of each DNA genome [24]. Similarly, transposable elements present dinucleotide compositions that are distinguishable from those observed in host genes [25]. Indeed, in *A. thaliana*, we found that the frequency of most dinucleotides is strikingly distinguishable between the repeatome and the CDS space (Figure 5). As compared to the latter, the frequency of all dinucleotides that contain only adenine and thymine is higher in repeated sequences whereas the frequency of dinucleotides that contain cytosine and/or guanine is lower except for the dinucleotides AC and GT which frequencies are indistinguishable between host genes and repeats. Interestingly, we found that the sequences corresponding to the additional annotations from the reiterative runs present a compositional bias that is reminiscent of the one obtained for repeated sequences although tending towards a gain in the differences with the one observed for host gene CDS (Figure 5).

Together, our results advocate that such reiterative approach is valuable for a thorough annotation of eukaryotic repeatomes while using a single program for the generation of consensus sequences. Furthermore, our results propose that the reiterative strategy also enables the identification of more ancient repeated and repeat-derived sequences that were out of reach using consensus sequences as probes but that become detectable using the enhanced repeatome diversity residing in a pool of known genomic copies.

### Performance of relaxed de novo approaches

Until here, we have employed different approaches that begun with the *de novo* construction of consensus repeats using conservative parameters. The similarity-based approaches such as TEdenovo have the advantage to be extensively tunable since the initial step consists in the identification of high-scoring pairs by all-by-all genome comparison. Intuitively, we reasoned that using relaxed parameters for HSP detection could lead to the construction of libraries of consensus sequences that may represent a wider, or to some extent different, diversity of the repeatome. Therefore in another attempt to uncover deep layers of the *A. thaliana* repeatome, we have used relaxed parameters for HSP detection with TEdenovo. Although relaxed, we chose the parameters as to detect highly significant matches. While the default filters for HSP detection are set to 90% identity and E-value < 1e-300, we performed two runs: one called “TEdenovo_cool” with these parameters set at 85% and 1e-50, and the second called “TEdenovo_soft” with parameters set at 80% and 1e-20, respectively. These latter values mean that we look for HSPs that are 80% identical with a score expected to be observed fewer than 1e-20 times in a random dataset. The HSPs detected were further processed with the TEdenovo pipeline.

**Figure 6:**
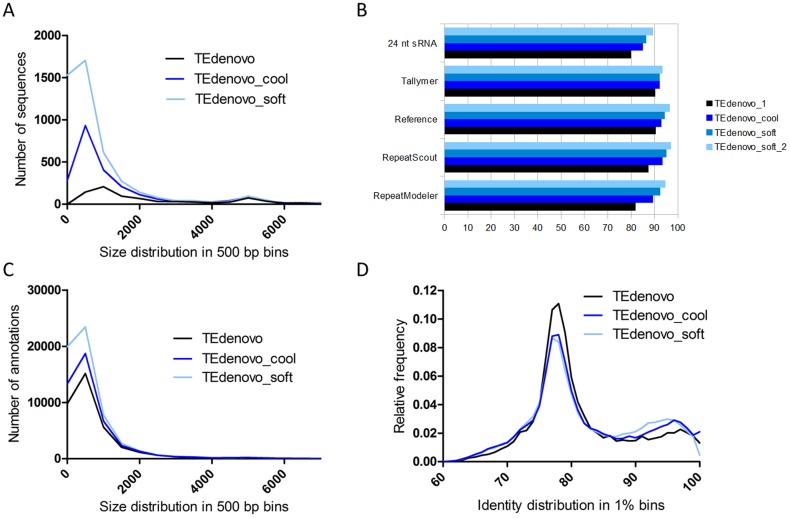
Relaxed parameters, ample effects. (A) Distribution in 500 bp bins of the size of the consensus sequences obtained using different parameters with TEdenovo. (B) Coverage of different indicators of sensitivity by annotations obtained using the relaxed approaches. (C) Distribution in 500 bp bins of the size of the annotations obtained using consensus sequences from the relaxed approaches. (D) Distribution in 1% bins of the identity values between genomic copies and consensus sequences from the relaxed approach.

These relaxed parameters were found to have a dramatic impact on the size of the library with the TEdenovo_cool and TEdenovo_soft libraries comprising 2,466 and 4,775 consensus sequences, respectively, as compared to 884 with regular TEdenovo. Qualitatively, the size distribution of the consensus from the TEdenovo_cool library shows a significant increase in the number of smaller sequences, while the number of large sequences remains stable as compared to regular TEdenovo (Figure 6A). This trend is accentuated with the TEdenovo_soft library. This suggests that relaxing the parameters for HSP detection has enabled detecting short HSPs that contributed to the generation of consensus libraries comprising an enriched complexity of short repeats as compared to default parameters. Because we use relaxed parameters, we expect to increase the contribution of host gene-derived consensus sequences in our library. Indeed, looking for putatively older duplication events could in theory identify more ancient amplifications of specific gene families as well as an increased number of conserved protein-coding domains. These, however, cannot be considered as false positives methodologically speaking.

Both relaxed libraries were used separately as inputs to annotate the *A. thaliana* genome. Overall, the TEdenovo_cool and TEdenovo_soft annotations cover 33.4 and 38.1 Mb of the genome to which CDS contribute 12.5 and 17.7%, respectively. The sensitivity of these annotations also fairly exceeds the one obtained with regular TEdenovo as measured following the indicators described above. For example, TEdenovo_soft covers as much as 94.5% of the reference set, and 95.3% of the RepeatScout annotations (Figure 6B). It thus appears that the TEdenovo libraries built using relaxed parameters hold a greater diversity of the *A. thaliana* repeatome as compared to those generated using default settings. We have assessed to what extent these annotations can cross-validate the annotations from our previous attempts. We found that the TEdenovo_soft annotations cover 83% of the TEdenovo_4 set. Reciprocally, the latter set covers 81% of the former (data not shown). In order to measure more specifically the overlaps with repeats that are confidently non- host gene-derived, we narrowed our comparisons to the relaxed annotations that do not overlap with CDS positions and found that annotation sets from the reiterative approach cover 84–94% of the non-CDS TEdenovo_soft annotations (28.4 Mb) (Figure S2). In a greedy tentative, we found that running a second iteration as previously with the TEdenovo_soft annotations (referred to as TEdenovo_soft _2) significantly increases the coverage of our proxies (Figure 6B) and enables to detect 92% of the TEdenovo_4 annotations (data not shown), thereby suggesting a limited contribution of false-positive annotations using the re-iterative approach.

**Figure 7:**
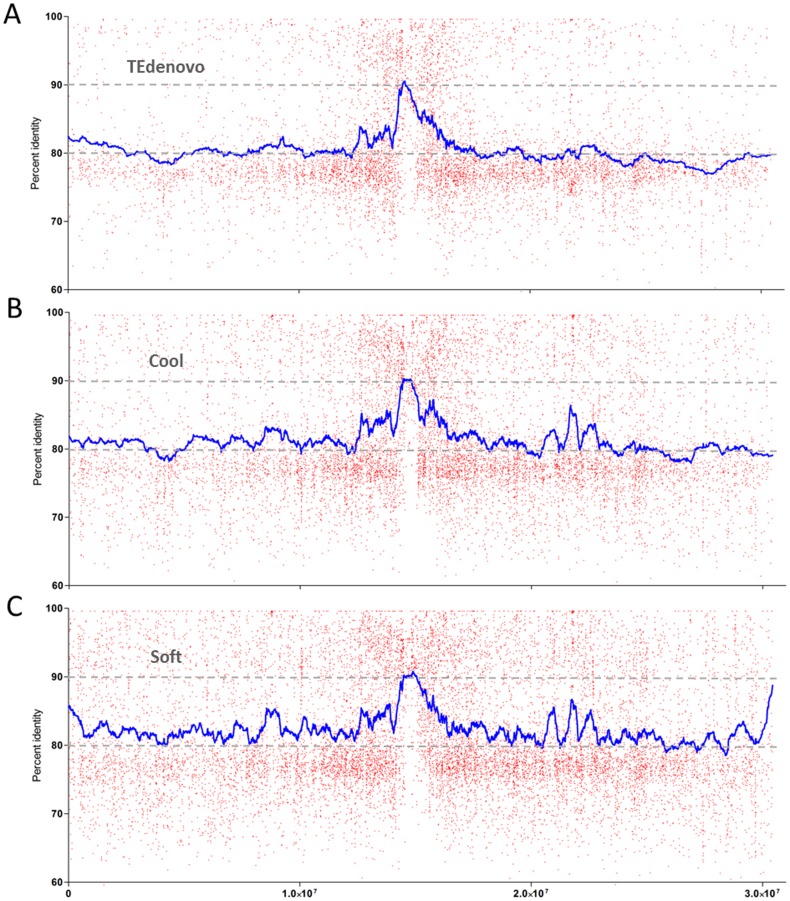
Landscape of identity values. Plot and smoothed curve (100 neighbors) of the identities between genomic copies and consensus sequences from TEdenovo (A), TEdenovo_cool (B), and TEdenovo_soft (C) along *A. thaliana* chromosome 1. Because identity values are not equally spaced, the smoothing is approximate and is not strictly “Savistsky-Golay” smoothing.

When measuring the size of the copies obtained using the relaxed libraries, we found that these were enriched in smaller fragments, especially in the 500 bp-1 kb range (Figure 6C). Interestingly, the distributions of the identities between consensus sequences and genomic copies of the TEdenovo_cool and TEdenovo_soft annotations present an increased frequency of high (over 85%) identity values as compared to TEdenovo_1 (Figure 6D). We further assessed the distribution of the identities between consensus sequences and genomic copies along the *A. thaliana* chromosome 1 in order to visualize whether the shift in frequency profile is to be attributed to randomly distributed annotations or if specific regions are affected. As described previously (Maumus and Quesneville, unpublished data), the highest identity values from the standard TEdenovo annotations are skewed towards pericentromeric regions (Figure 7A). Interestingly, while we observe an overall shift towards higher identity values in the distribution of the TEdenovo_cool annotations, this effect is of heterogeneous magnitude. For instance, along the chromosome 1, the pericentromeric regions appear to be significantly affected as well as a large region defined by three contiguous peaks located around 20–23 Mb (Figure 7B). With the TEdenovo_soft annotations, the distribution of high identity values appears to be less region-specific with an overall apparently more diffuse signal as compared to TEdenovo_cool (Figure 7C). This result may be explained in part by the greater host gene contamination in the TEdenovo_soft annotations. Specifically though, the TEdenovo_soft annotations present even greater peaks of high identity values in the region located at 20–23 Mb as well as peaks in the telomeric regions of chromosome 1.

By identifying and reciprocally validating most of the annotations obtained through non conservative approaches above, these relaxed approaches therefore tend to confirm that a substantial amount of the *A. thaliana* genomic dark matter is of repetitive origin. Our results also enable to reveal the peculiar nature of specific regions on chromosome 1 which evolutionary histories will deserve investigation.

### Validation using information fetched from close relatives

During periods of genome down-sizing with low TE activity as in *A. thaliana* [26] [27] [28] [29] [30], severe sequence decay and physical genome purification can crumble or abolish the repetitive nature of ancient repeats. However, after the separation from a common ancestor, repeat families have different fate in different species so that some ancestral repeat families may be differently preserved within a group of species. Therefore, some repeat families that are difficult to detect in *A. thaliana* using conservative methods may be better recognized by libraries of consensus sequences established from the genomes of closely related species. As introduced elsewhere (Maumus and Quesneville, unpublished data), we tried to take advantage of information fetched from the genome of six *A. thaliana* relatives (family *Brassicaceae*) that diverged *c*. 5–40 million years ago (mya) [31] [32]: *Arabidopsis lyrata*, *Capsella rubella*, *Arabis alpina* (courtesy of Dr. George Coupland), *Brassica rapa*, *Thellungiella salsuginea* (formerly *Thellungiella halophila*), and *Schrenkiella parvulum* (formerly *Thellungiella parvula*) [29] [30] [33] [34] [35] (Figure S3A). Additionally, besides the genome from the reference *A. thaliana* accession Col-0, we also acquired information from the genome assembly of four other ecotypes (Ler-1, Kro-0, Bur-0, and C24) [36]. For each of these genomes including Col-0, we obtained a library of consensus sequences using TEdenovo that we all combined into a “*Brassicaceae*” library (Figure S3A).

Incidentally, this library provides a highly sensitive annotation of the *A. thaliana* repeatome (Maumus and Quesneville, unpublished data). For instance, the *Brassicaceae* annotations cover more than 90% of the 24-nt sRNA map as compared to 80% with the TEdenovo_1 annotations. In the same vein, the *Brassicaceae* annotations cover nearly 97% of the reference annotations as compared to 90% with the TEdenovo_1 annotations (Maumus and Quesneville, unpublished data). Remarkably, we found that the *Brassicaceae* annotations also cover 89% (33.2 Mb) of the TEdonovo_4 annotations, suggesting high specificity of the reiterative approach (data not shown). Significantly as well, we measured that the *Brassicaceae* annotations further cover 94% of the TEdenovo_cool annotations as well as 89 and 86% of the TEdenovo_soft and TEdenovo_soft_2 annotations, respectively, again supposing limited false positive rate in the relaxed approaches described above (data not shown). More specifically, we found that the *Brassicaceae* annotations that do not overlap with CDS features also cover 91% of the non-CDS TEdenovo_soft annotations (Figure S3B). Following the differential retention of ancestral repeat families in modern genomes, the high coverage achieved by the *Brassicaceae* annotations over the relaxed and reiterative ones suggests that these latter are enriched with ancestral repeats. Overall, the extensive cross-validation of non-conservative approaches with the *Brassicaceae* annotations further illustrates the relevance of the usage of an evolutionary diverse repeat library for genome annotation and provides evidence that ancestral repeats can also be unveiled using the reiterative and relaxed methods presented above.

### Attempt towards a thorough and conservative annotation

**Figure 8:**
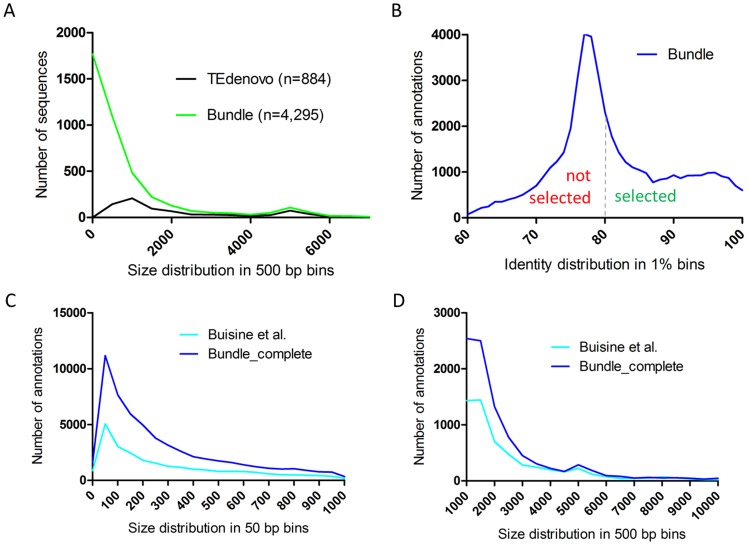
Deep and conservative annotation of the *A. thaliana* repeatome. (A) Distribution in 500 bp bins of the size of the non-redundant sequences from the “Bundle” library. (B) Distribution in 1% bins of the identity values between genomic copies and consensus sequences from the “Bundle” library. The dashed line indicates the 80% identity threshold applied to select the copies that were used to run the “Bundle_2” annotation. (C–D) Distribution in 50 bp bins for short segments (C) and in 500 bp bins for long segments (D), of the size of the repeat annotations from TAIR10 [37] and from the Bundle_complete annotation.

We try to learn from the results of the attempts described above. We found that (i) the libraries from the three programs RM, RS, and TEdenovo are complementary for a thorough annotation by providing a diversified repeatome seed (ii) the iterative annotation process enables to substantially increase the sensitivity of genome masking (iii) a substantial fraction of the *A. thaliana* dark matter is likely to be repeated or repeat-derived, that could not be detected using standard *de novo* repeat-finding approaches. Following these statements, we try to generate a deep and conservative annotation of the *A. thaliana* repeatome. While the sensitivity of the set comprising the TEdenovo, RS, and RM annotations is extensive, their combination would result in abundantly overlapping features; or generating a non-redundant set would yield highly fragmented annotations and would demand to apply priority rules. To palliate this problem and to take advantage of a maximum of evidence, we have combined the TEdenovo, RS, and RM libraries, as well as the reference TE sequences and removed redundancy from this set (see Methods, Figure 8A) in order to generate a “Bundle” library that we used to annotate the *A. thaliana* genome. Following statement ii, we performed a second iteration (Bundle_2) using the copies from the first round as input library. In order to be empirically conservative, only the copies with size > = 200 bp that are less than 20% divergent from their respective consensus sequences were selected as probes for this second iteration (Figure 8B). As an empirically reassuring indicator of conservativeness, running a third iteration using similar filters on the copies from the second round did not increase genome coverage. We have further complemented the Bundle_2 annotations by incorporating the annotations from the Bundle iteration that are absent in Bundle_2. This annotation set (referred to as Bundle_complete), obtained using a combination of conservative approaches, covers 37.7 Mb of the Col-0 genome to which CDS contribute 4.8 Mb. Remarkably, the Bundle_complete annotation covers 88% of our 24-nt sRNA map. It also covers 97% of the current TE annotation available for TAIR10 [37] (totaling 23.3 Mb) indicating that the Bundle_complete annotation comprises the vast majority of previously annotated repeats in *A. thaliana*. In contrast, we observe 61% of reciprocal coverage. The Bundle_complete annotation comprises about twice more annotations as compared to the existing TAIR10 TE annotation (66,259 versus 31,189). While, as anticipated, the Bundle_complete annotation comprises an increased number of short annotations (length < = 1 kb) as compared to the present TAIR10 annotation (Figure 8C), it also includes a higher number of long features (length > 1 kb) (Figure 8D). We wish that this repeat map, as well as those described above, will prove useful for a variety of genome and epigenome studies.

### Unveiling the repetitive origin of genomic dark matter using P-clouds

Above, we have annotated the *A. thaliana* genome by comparing it to libraries of consensus sequences using local alignment programs embedded in the REPET package. However, because repeated and repeat-derived sequences become more and more mutated and fragmented over time, the alignment-based approaches may be little efficient to detect the oldest repeat remnants. Nevertheless, the decay of ancient repeated sequences would have produced a significant amount of short, (nearly) identical repeats. The physical clustering of such high frequency oligos in consecutive or near consecutive regions on the chromosomes is then likely to testify the presence of repeat remnants. The P-clouds package was conceived following this postulate and enabled to identify that the human repeatome may contribute two-thirds of the human genome as compared to the 45–50% detected using alignment-based programs [18] [38]. P-clouds identifies repeated k-mers in a DNA sequence and groups into “clouds” closely-related oligos that occur, as a group, more often than predicted by chance. The projection on the genome of k-mers that were grouped into “clouds” is then computed and significant local density demarcates regions that are of putative repetitive origin.

**Figure 9:**
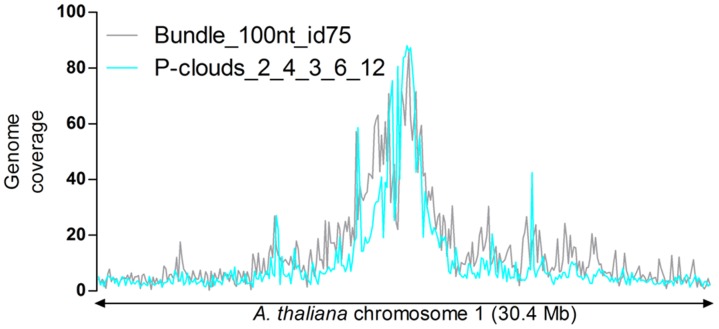
Repeat-specific P-clouds annotation. Density along *A. thaliana* chromosome 1 of the input data (“Bundle” filtered) and output of the P-clouds annotation.

Considering that a large fraction of the *A. thaliana* repeatome is rather ancient (Maumus and Quesneville, unpublished data), we assessed whether P-clouds would detect additional regions of putative repeated origin in the *A. thaliana* genome. We used the program following a ‘repeat-specific’ approach, similar to the one applied on the human genome [18], *i.e*. building ‘clouds’ of k-mers from copies detected with alignment-based approaches. From the “Bundle” annotation (first iteration), we extracted the copies of at least 100 bp and 75% identity to the cognate consensus sequence. We tried different parameters for clouds construction and determined a set that appeared empirically relevant (essentially by assessing true positive rate by comparison to the Bundle annotation and false positive rate by comparison to the set of host-gene CDS and by visualization of density along the chromosomes). P-clouds annotations covered 14 Mb of the *A. thaliana* genome. When mapping the density of P-clouds annotations along the chromosome 1, we found that it correlates significantly with the density of the input data (Figure 9). Unexpectedly, we found that host gene CDS contribute 17% (2.4 Mb) of the P-clouds space. It remains to be determined whether this reflects a contamination due to the presence of host genes in the input or if it indicates the significant incorporation of other repeat types within CDS during evolution.

We determined the regions demarcated by P-clouds that were newly identified as compared to the Bundle_complete annotation and found 6 Mb of P-clouds-specific regions. Newly detected segments were more abundant in the pericentromeric regions as compared to chromosome arms (Figure S4–S8). Interestingly, for chromosomes 3, 4, and 5, the density of the new P-clouds segments appears especially high on one specific side of the pericentromeric region, suggesting asymmetric composition. Cumulatively, the Bunble_complete and P-clouds annotations cover 89% of the 24nt sRNA set and represents 43.7 Mb, *i.e.* over one third of the *A. thaliana* genome.

## Discussion

The work presented here illustrates that different *de novo* repeat finding programs are capable to seize slightly different fragrances of the repeatome. Together, the consensus sequences built by these programs appear to hold an increased diversity of the *A. thaliana* repeatome as compared to libraries from a single program. Empirically, the extent of this complementarity is likely to correlate with the complexity of the repeatome assessed. In reducing genomes with low TE activity such as *A. thaliana* [26] [27] [29], aging copies become increasingly difficult to detect due to sequence divergence and loss of repetitive nature. In contrast, inflating genomes with high TE activity accumulate highly similar sequences that in addition provide templates for DNA loss mediated by illegitimate recombination [39]. The resulting turnover is likely to speed-up the clearance of old repeats, thereby diminishing repeatome complexity and facilitating repeatome detection. It will be of interest to test the performance of such combined approaches on genomes with contrasting junk DNA composition.

The reiterative approach is suggestive of an archeology toolkit that would enable to scrape successive layers containing traces of more ancient repeated and repeat-derived sequences. The profiles of the dinucleotide frequencies from the consecutive iterations are typical of the repeatome and tend towards enrichment in dinucleotides comprising only A and T which is consistent with the effect of spontaneous deamination of methylcytosines [40] [41]. Incidentally though, the dinucleotides AC and GT appear at the same frequency in the CDS and repeat sets (Figure 5). This may echo with the observation that, while methylation in the CG context is preponderant in *Arabidopsis* TEs compared to CHG and CHH (where H stands for A, T and C), the lowest CG methylation levels are highly enriched for the palindromic sequence ACGT [42]. In the same vein, lowering the stringency of the parameters for HSP detection with TEdenovo enables to build consensus libraries enclosing an increased diversity of the repeatome through the initial detection of more diverged copies as compared to conservative approaches. This method also appears especially relevant for detecting repeatome layers corresponding to remnants from ancestral copies.

Over time, copy divergence accompanied by insertions and deletions can lead to extensive fragmentation of the original sequences to such an extent that they can intrinsically not be detected using consensus-based alignments. The principle of P-clouds enables to search for regions comprising such locally crumbled information. Indeed, we show that, as with the human genome [18], P-clouds enables to identify regions that were not identified using methods invoking local alignments. The distribution of these annotations along the chromosomes presents highest densities in pericentromeric regions, which is in agreement with putative repetitive origin thereby suggesting that this approach permitted to unveil one more layer of the ancestral repeatome.

All the annotations generated during this work are visible on a genome browser at https://urgi.versailles.inra.fr/gb2/gbrowse/tairv10_pub_TEs/ where tracks can also be downloaded. We provide a snapshot of this genome browser in Figure S9 representing an overview of our work in the context of a pericentromeric region. As shown in this figure, the new annotations cover several previously not annotated regions (i.e. previously genomic dark matter). The majority of these new annotations are supported by 24-nt small RNA coverage. Interestingly, several new annotations also cover a predicted host gene (AT1G43760). Sequence analysis shows that this gene model was built on the sequence of a L1-type non-LTR retrotransposon (data not shown). This predicted gene is also densely covered by 24-nt sRNAs. Together, this snapshot illustrates the relevance of our work and we anticipate that these annotations will be considered as valuable resources for future biological and evolutionary studies.

Overall, besides proposing new approaches and providing significant knowledge to our understanding of the *A. thaliana* genome composition, our work emphasizes the idea that genomic dark matter and repeated sequences may, to a large extent, represent the two extremes of a continuum. Indeed, by looking for repetitive and repeat-derived elements using non-conservative approaches, we have been annotating a substantial amount of genome fragments that never were before. Our results suggest that we may have reached the limit of detectable ancestral information so that we speculate that sequences that could be attributed a repetitive origin today may only be the tip a melting junk DNA iceberg.

## Methods

### Datasets

Genome sequences were obtained from the following sources: *A. thaliana* ecotype Col-0 (TAIR10 release) (http://www.phytozome.com/arabidopsis.php); *A. thaliana* ecotypes Ler, Kro, Bur, and C24 (http://www.1001genomes.org/); *A. lyrata* (http://www.phytozome.com/alyrata.php); *C. rubella* (http://www.phytozome.com/capsella.php); *A. alpina* (preliminary release, Courtesy of George Coupland); *T. parvula* (v2.0, http://thellungiella.org/data/); *T. halophila* (http://www.phytozome.com/thellungiella.php); *B. rapa* (v1.2, http://www.phytozome.com/napacabbage.php). The 24-nt small RNA map used in this work corresponds to the 24 bp annotations extracted from the TAIR7 dataset GSM277608 (http://www.ncbi.nlm.nih.gov/geo/) and transposed to the TAIR10 assembly. The library of reference sequences was generated by compiling the *A. thaliana* reference sequences from Repbase [43] and a set of manually curated consensus sequences [37] followed by redundancy removal (with thresholds of 95% identity and 98% coverage). The reference annotations were obtained by using this library as input for the TEannot pipeline (see below). This library was appended to the *Brassicaceae* library in order to perform the group approach except for the *A. thaliana* annotation in order not to introduce knowledge-based bias in this comparative analysis. The CDS dataset corresponds to the positions of the CDS from host gene models in TAIR10 (ftp://ftp.arabidopsis.org/Maps/gbrowse_data/TAIR10/), *i.e*. excluding “transposable element” genes.

### Genome annotation

Some programs such as Tallymer and TRF perform both repeat detection and genome annotation. In contrast, the consensus sequences that are built by RepeatScout, RepeatModeler, and TEdenovo must be provided as inputs to homology-based genome annotation programs such as RepeatMasker [44], CENSOR [45], and BLASTER [46]. The TEannot pipeline from the REPET package uses a wrapper that launches and computes results from these three programs. Using a library of *A. thaliana* reference repeats (see above) we found that, as previously shown [47], TEannot provides increased sensitivity compared to the results obtained using RepeatMasker alone (Figure S10A). Importantly, the annotations obtained with TEannot were not skewed towards shorter sizes as compared to those obtained with RepeatMasker (Figure S10B) showing that the supplementary annotations cannot be accounted by a bias towards detecting shorter segments. Instead, TEannot yields longer annotations on average, as compared to RepeatMasker alone (SUPTABLE1). Therefore we have employed TEannot as masking tool in all the experiments described in this work.

### Estimation of host gene fraction of the repeatome

We have characterized the composition of the RepeatScout, RepeatModeler, and TEdenovo annotations in order to distinguish the repeats that putatively correspond to host genes from those that do not. Indeed, we know from experience that in most genomes, conservative *de novo* repeat identification is likely to detect freshly duplicated host genes and highly conserved protein domains because they do are repeated or contain repeated segments. Genes can also contain insertions of mobile elements, especially within introns. Thus, without manual examination, it can be difficult to discriminate if intronic repeats come from gene duplication or insertion of mobile elements. Nevertheless, following gene duplication, protein-coding sequences are more likely to be repeated than introns upon which a lesser selection pressure occurs, on average. Therefore as an indicator of the contribution of host gene-derived repeats in our annotations, we measure the fraction corresponding to the coding DNA sequences (CDS) only. As a result, we found that CDS contribute 6.6, 9.9 and 11.1% of the TEdenovo, RS and RM annotations, respectively, while the combined set is made of 12.8% of CDS. This rate has to be compared to the composition of the compact Arabidopsis genome to which CDS contribute 28.1%. Correlatively, the TEdenovo, RS and RM annotations cover 6–9% of the CDS space, while the combined set attains 13.5% coverage. Hence, using conservative settings, it appears that about 10% of the CDS space is repeated. The absolute CDS contribution to our set of annotations is illustrated in Figure 4.

### *De novo* identification of repeated sequences

Tallymer was run with parameters searching for 20-mers with at least 4 occurrences. TRF was run with the following set of parameters: 2, 10, 10, 80, 10, 24, 2000. RepeatScout was launched using default settings except extension parameter (stopafter) which was set at 500. The seed size as determined by the program was 15 bp for the *A. thaliana* genome. RepeatModeler was employed with default settings except that, again, the RepeatScout extension parameter (stopafter) was set at 500. TEdenovo from the REPET pipeline (v2.0) was run with default parameters. Consensus sequences derived from LTR Harvest predictions were retained only when they presented pfam domains typical of LTR retrotransposon. The parameters for HSP detection with TEdenovo were modified to perform the “cool” run (Evalue: 1e-50 and Identity = 85) and the “soft” run (Evalue: 1e-20 and Identity = 80). P-clouds was run using 15-mers and the suite of parameters 2, 4, 3, 6, 12.

### Data processing and filtering

The annotations covering less than 100 bp were discarded in order to draw the plots showing the frequency distribution of identity between consensus sequences and copies, the plots showing identity values between consensus sequences and copies along genome sequences, and the plots showing the distribution of copy sizes. The TEannot pipeline from the REPET package performs a “long join” procedure in order to connect fragments that may have been interrupted by a relatively recent insertion event. The sizes of the annotations presented in this work correspond to the results prior this post processing reconstruction step whereas the annotation files loaded on the genome browser correspond to results that benefit the long join procedure. To generate the Bundle library, redundancy in the combined set of consensus sequences from TEdenovo, RepeatScout, RepeatModeler, and reference sequences was filtered using all-by-all blast comparisons with thresholds of 95% identity and 98% length, resulting in 4,295 selected sequences. Coverages, differences and overlaps between datasets were computed using the S-MART suite [48]. Dinucleotide composition was calculated using compseq from the EMBOSS package.

## Supporting Information

Figure S1 **Circular comparative repeat densities.** Outer to inner: *A. thaliana* chromosomes (scale unit  = 1 Mb); repeat density from TEdenovo (black), repeat density from RepeatModeler (green), repeat density from RepeatScout (red), CDS density (blue); chromosome number. For each density track, density is calculated in 100 kb windows with 10 kb overlap between consecutive windows and the scale ranges from 0 to 100% genome coverage.
(TIF)

Figure S2 **Validation of the relaxed annotations.** Coverage of the TEdenovo_soft annotations that do not overlap with *A. thaliana* CDS by different sets of annotations.
(TIF)

Figure S3 **Performance and effects of the group approach.** (A) Cladogram representing the phylogenetic relationships between the *Brassicaceae* species used to construct the *Brassicaceae* library with TEdenovo. Arrows indicate branching dates as approximated from previous studies [31] [32]. (B) Coverage of the *Brassicaceae* annotations that do not overlap with *A. thaliana* CDS by different sets of annotations.
(TIF)

Figure S4 **Deep repeat landscape along** ***A. thaliana*** **chromosome 1.** Density of the annotations from TEdenovo, Bundle_complete, Bundle_complete plus P-clouds, and the P-clouds-specific set (Delta) along chromosome 1.
(TIF)

Figure S5 **Deep repeat landscape along** ***A. thaliana*** **chromosome 2.** Density of the annotations from TEdenovo, Bundle_complete, Bundle_complete plus P-clouds, and the P-clouds-specific set (Delta) along chromosome 2.
(TIF)

Figure S6 **Deep repeat landscape along** ***A. thaliana*** **chromosome 3.** Density of the annotations from TEdenovo, Bundle_complete, Bundle_complete plus P-clouds, and the P-clouds-specific set (Delta) along chromosome 3.
(TIF)

Figure S7 **Deep repeat landscape along** ***A. thaliana*** **chromosome 4.** Density of the annotations from TEdenovo, Bundle_complete, Bundle_complete plus P-clouds, and the P-clouds-specific set (Delta) along chromosome 4.
(TIF)

Figure S8 **Deep repeat landscape along** ***A. thaliana*** **chromosome 5.** Density of the annotations from TEdenovo, Bundle_complete, Bundle_complete plus P-clouds, and the P-clouds-specific set (Delta) along chromosome 5.
(TIF)

Figure S9 **Genome browser with new annotations.** Snapshop of the *A. thaliana* genome browser showing a region located on chromosome 1 at positions 16,528-16,560 kb. The name of each track appears in bold black text on the left side.
(TIF)

Figure S10 **Comparative genome masking with TEannot versus RepeatMasker alone.** (A) Coverage of the *A. thaliana* genome obtained with the TEannot pipeline and RepeatMasker softwares using the reference library. (B) Distribution in 500 bp bins of the size of the reference annotations with respect to the masking software used.
(TIF)
