# Supplementary figures and images for: Deep Investigation of Arabidopsis thaliana Junk DNA Reveals a Continuum between Repetitive Elements and Genomic Dark Matter
Source: PLoS One. 2014 Apr 7;9(4):e94101. doi: 10.1371/journal.pone.0094101 (PMC3978025; doi:10.1371/journal.pone.0094101)

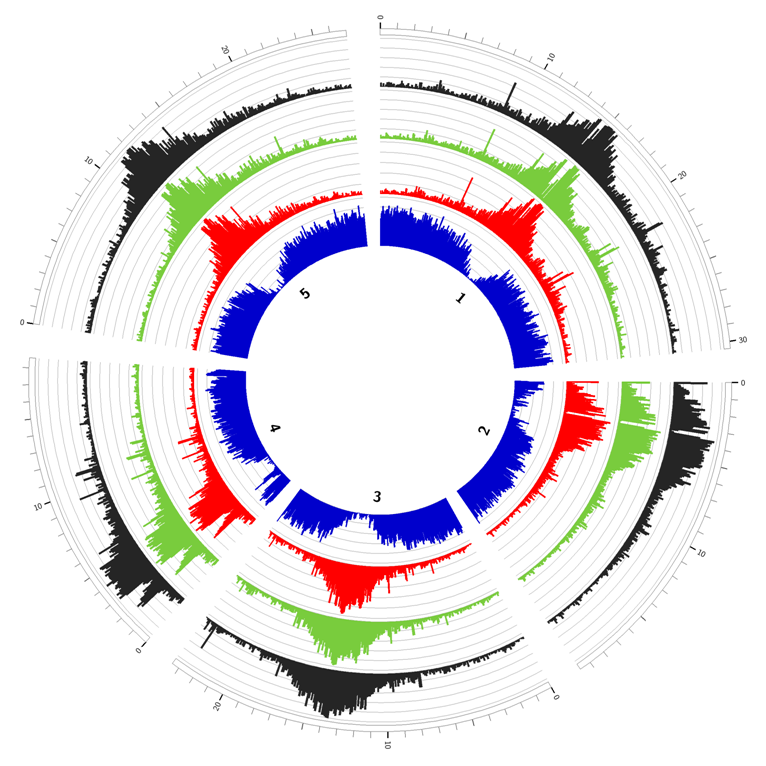

Supplement: Figure S1 — Circular comparative repeat densities. Outer to inner: A. thaliana chromosomes (scale unit = 1 Mb); repeat density from TEdenovo (black), repeat density from RepeatModeler (green), repeat density from RepeatScout (red), CDS density (blue); chromosome number. For each density track, density is calculated in 100 kb windows with 10 kb overlap between consecutive windows and the scale ranges from 0 to 100% genome coverage. (TIF) [file pone.0094101.s001.tif]

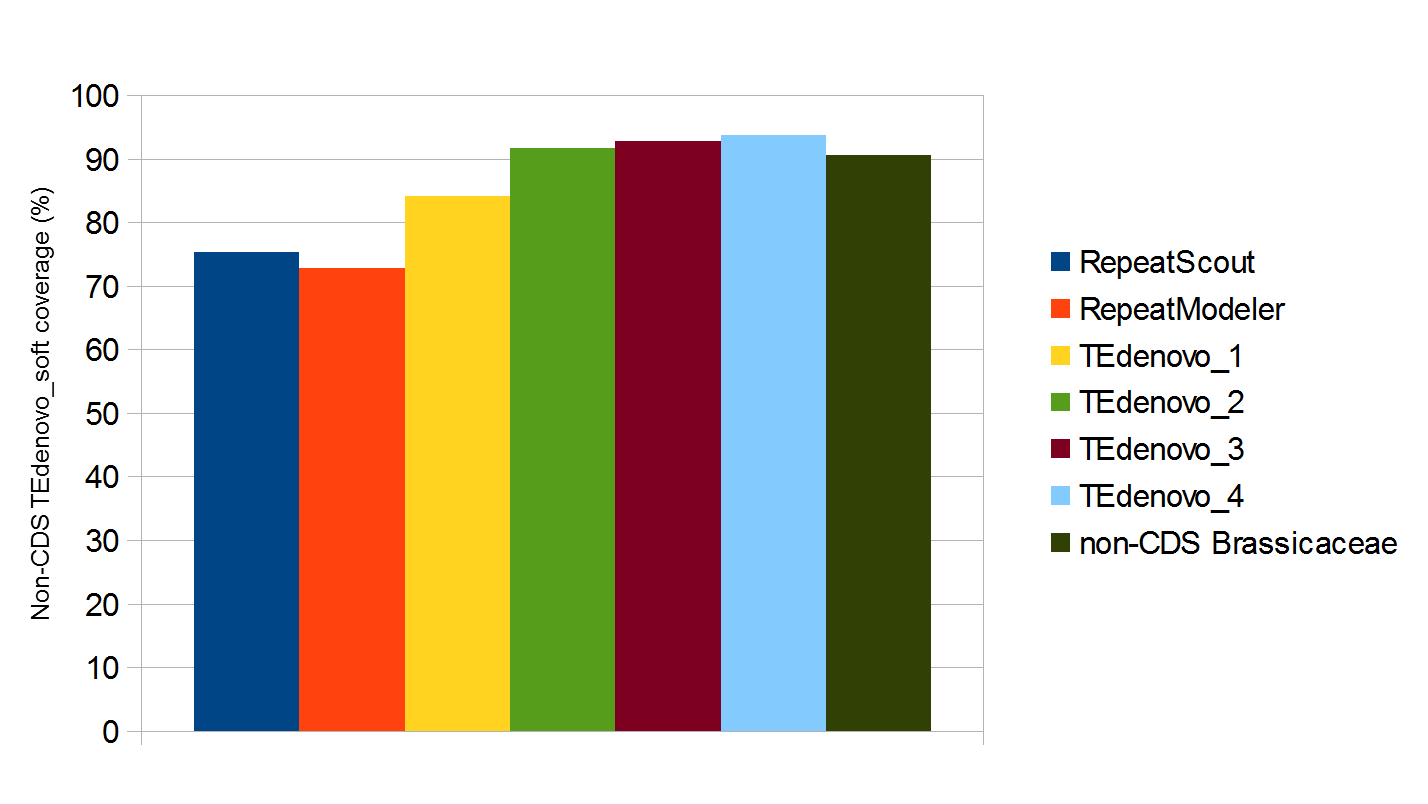

Supplement: Figure S2 — Validation of the relaxed annotations. Coverage of the TEdenovo_soft annotations that do not overlap with A. thaliana CDS by different sets of annotations. (TIF) [file pone.0094101.s002.tif]

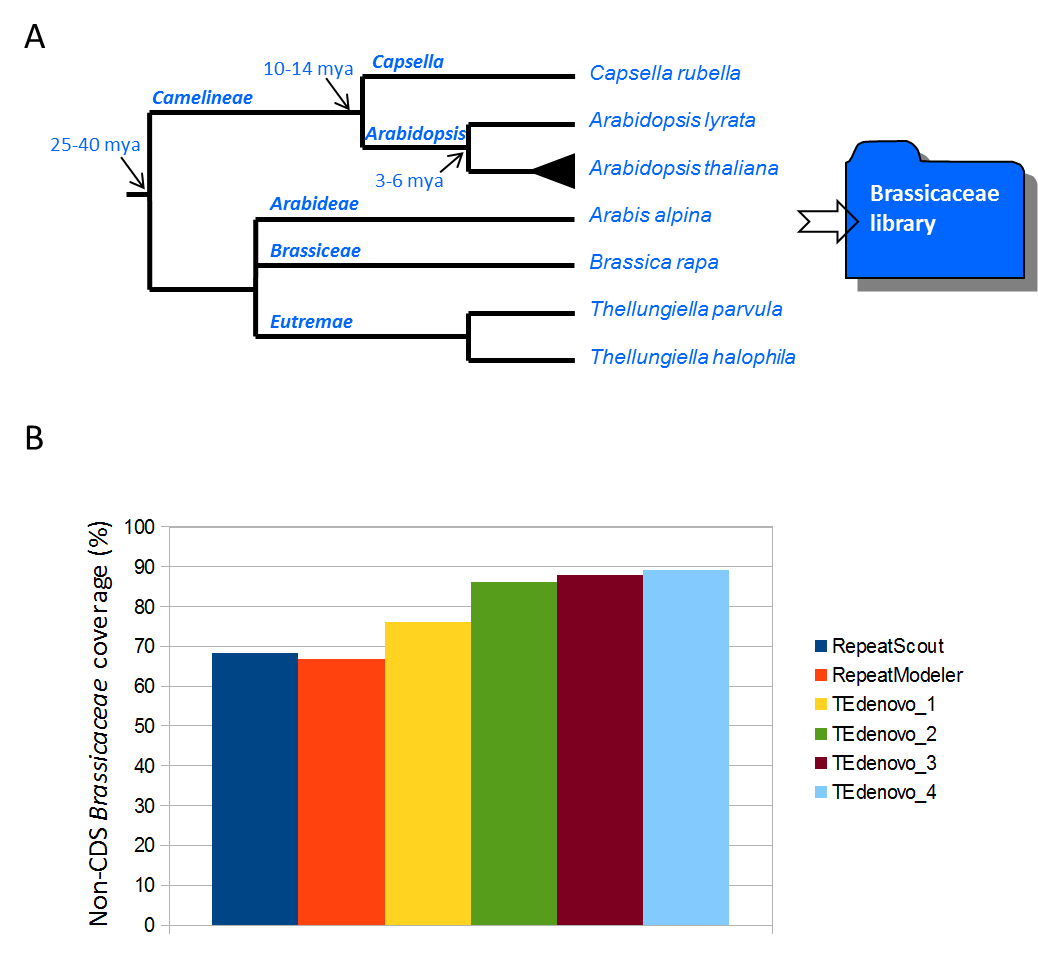

Supplement: Figure S3 — Performance and effects of the group approach. (A) Cladogram representing the phylogenetic relationships between the Brassicaceae species used to construct the Brassicaceae library with TEdenovo. Arrows indicate branching dates as approximated from previous studies [31] [32]. (B) Coverage of the Brassicaceae annotations that do not overlap with A. thaliana CDS by different sets of annotations. (TIF) [file pone.0094101.s003.tif]

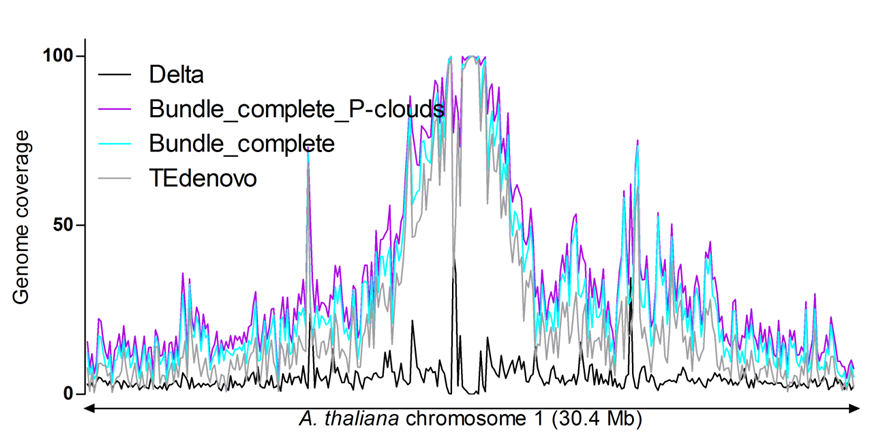

Supplement: Figure S4 — Deep repeat landscape along A. thaliana chromosome 1. Density of the annotations from TEdenovo, Bundle_complete, Bundle_complete plus P-clouds, and the P-clouds-specific set (Delta) along chromosome 1. (TIF) [file pone.0094101.s004.tif]

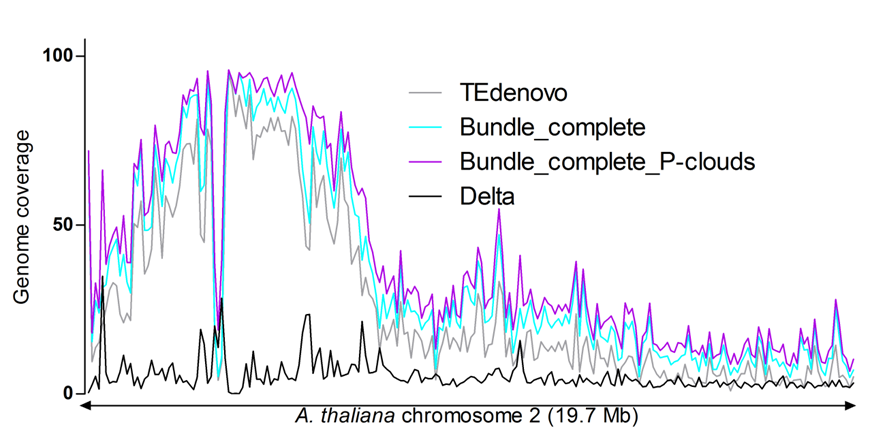

Supplement: Figure S5 — Deep repeat landscape along A. thaliana chromosome 2. Density of the annotations from TEdenovo, Bundle_complete, Bundle_complete plus P-clouds, and the P-clouds-specific set (Delta) along chromosome 2. (TIF) [file pone.0094101.s005.tif]

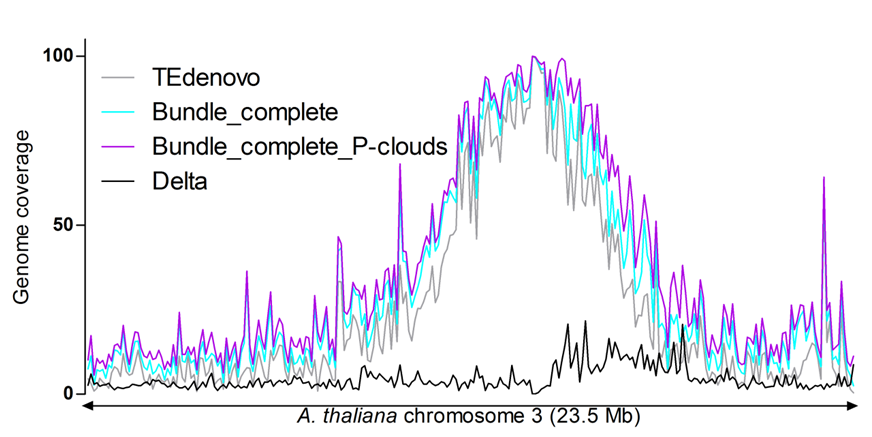

Supplement: Figure S6 — Deep repeat landscape along A. thaliana chromosome 3. Density of the annotations from TEdenovo, Bundle_complete, Bundle_complete plus P-clouds, and the P-clouds-specific set (Delta) along chromosome 3. (TIF) [file pone.0094101.s006.tif]

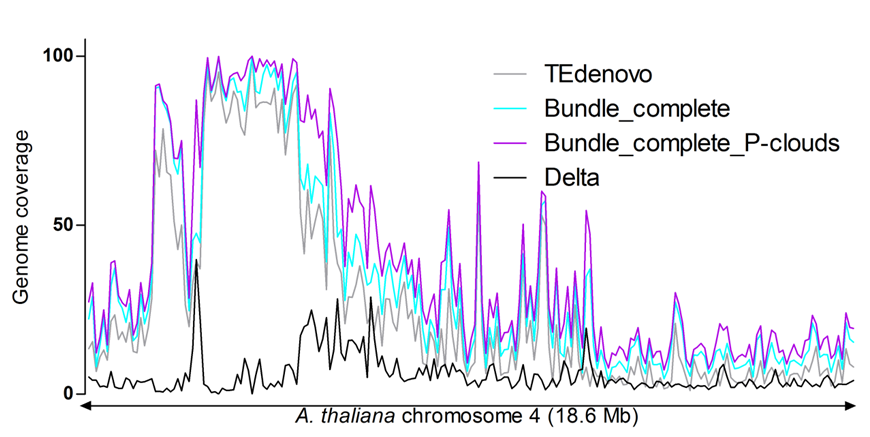

Supplement: Figure S7 — Deep repeat landscape along A. thaliana chromosome 4. Density of the annotations from TEdenovo, Bundle_complete, Bundle_complete plus P-clouds, and the P-clouds-specific set (Delta) along chromosome 4. (TIF) [file pone.0094101.s007.tif]

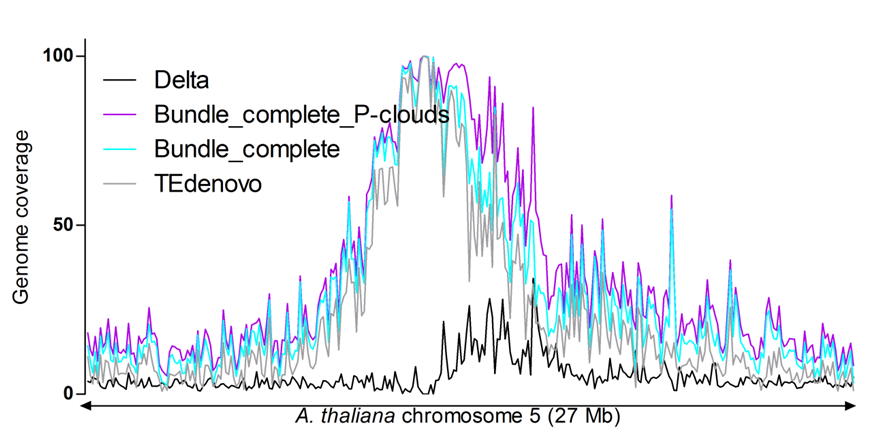

Supplement: Figure S8 — Deep repeat landscape along A. thaliana chromosome 5. Density of the annotations from TEdenovo, Bundle_complete, Bundle_complete plus P-clouds, and the P-clouds-specific set (Delta) along chromosome 5. (TIF) [file pone.0094101.s008.tif]

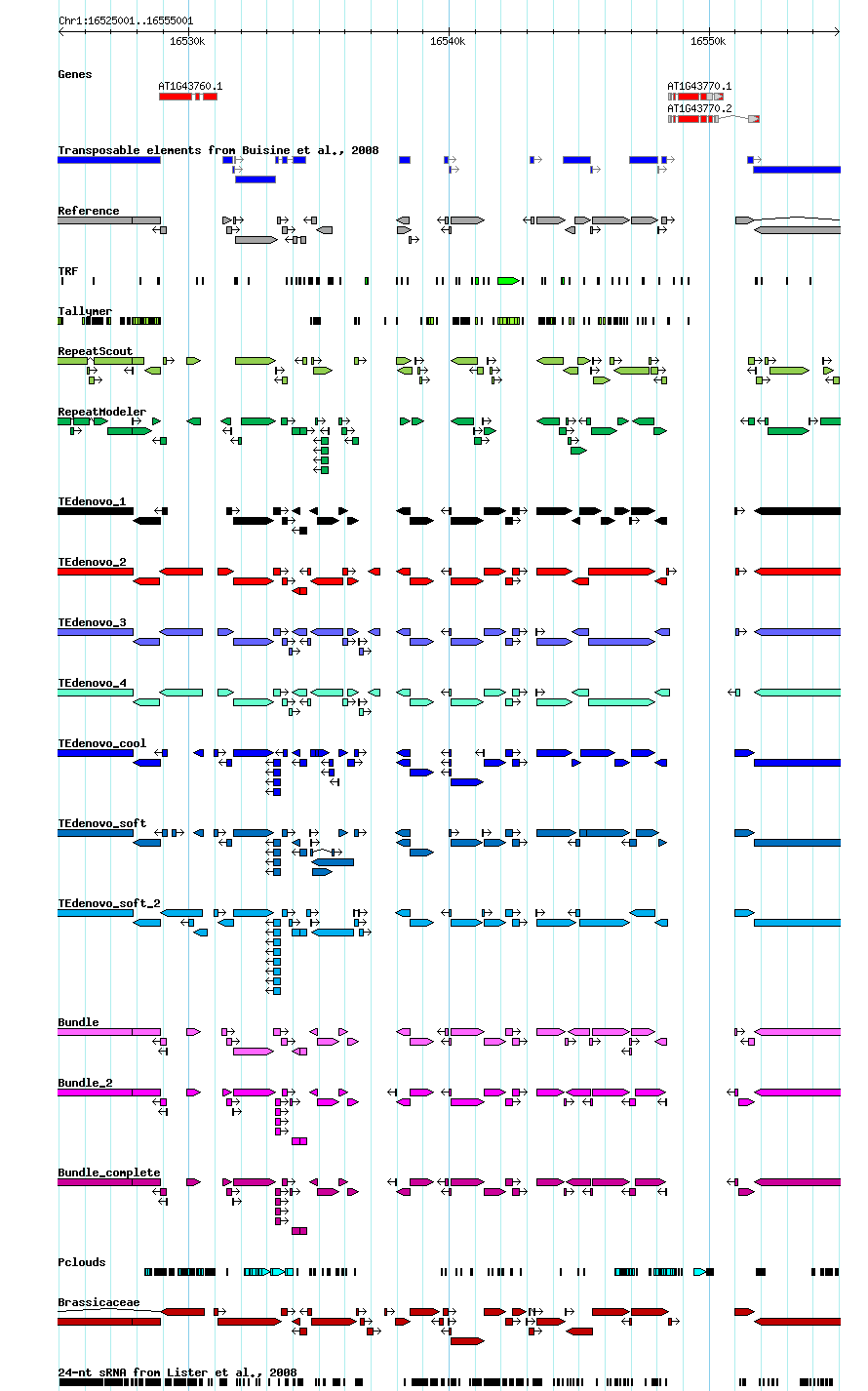

Supplement: Figure S9 — Genome browser with new annotations. Snapshop of the A. thaliana genome browser showing a region located on chromosome 1 at positions 16,528-16,560 kb. The name of each track appears in bold black text on the left side. (TIF) [file pone.0094101.s009.tif]

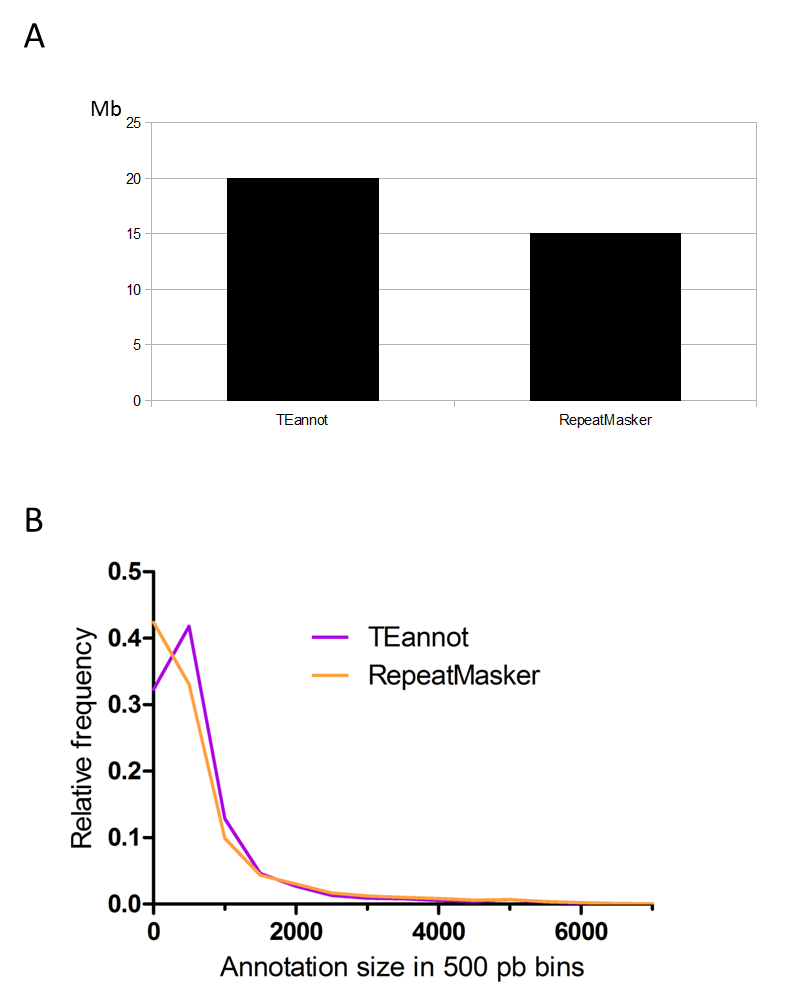

Supplement: Figure S10 — Comparative genome masking with TEannot versus RepeatMasker alone. (A) Coverage of the A. thaliana genome obtained with the TEannot pipeline and RepeatMasker softwares using the reference library. (B) Distribution in 500 bp bins of the size of the reference annotations with respect to the masking software used. (TIF) [file pone.0094101.s010.tif]
